# The Extracellular Matrix: Its Composition, Function, Remodeling, and Role in Tumorigenesis

**DOI:** 10.3390/biomimetics8020146

**Published:** 2023-04-05

**Authors:** Kevin Dzobo, Collet Dandara

**Affiliations:** 1 Medical Research Council, SA Wound Healing Unit, Hair and Skin Research Laboratory, Division of Dermatology, Department of Medicine, Groote Schuur Hospital, Faculty of Health Sciences, University of Cape Town, Anzio Road, Observatory, Cape Town 7925, South Africa; 2 Division of Human Genetics and Institute of Infectious Disease and Molecular Medicine, Department of Pathology, Faculty of Health Sciences, University of Cape Town, Anzio Road, Observatory, Cape Town 7925, South Africa; 3 The South African Medical Research Council-UCT Platform for Pharmacogenomics Research and Translation, Department of Pathology, Faculty of Health Sciences, University of Cape Town, Anzio Road, Observatory, Cape Town 7925, South Africa

**Keywords:** extracellular matrix, tissues, organs, development, tumor progression, collagens, fibronectin, integrins, metastasis, matrix metalloproteases, cell adhesion, signaling

## Abstract

The extracellular matrix (ECM) is a ubiquitous member of the body and is key to the maintenance of tissue and organ integrity. Initially thought to be a bystander in many cellular processes, the extracellular matrix has been shown to have diverse components that regulate and activate many cellular processes and ultimately influence cell phenotype. Importantly, the ECM’s composition, architecture, and stiffness/elasticity influence cellular phenotypes. Under normal conditions and during development, the synthesized ECM constantly undergoes degradation and remodeling processes via the action of matrix proteases that maintain tissue homeostasis. In many pathological conditions including fibrosis and cancer, ECM synthesis, remodeling, and degradation is dysregulated, causing its integrity to be altered. Both physical and chemical cues from the ECM are sensed via receptors including integrins and play key roles in driving cellular proliferation and differentiation and in the progression of various diseases such as cancers. Advances in ‘omics’ technologies have seen an increase in studies focusing on bidirectional cell–matrix interactions, and here, we highlight the emerging knowledge on the role played by the ECM during normal development and in pathological conditions. This review summarizes current ECM-targeted therapies that can modify ECM tumors to overcome drug resistance and better cancer treatment.

## 1. Introduction

Tissues and organs in the human body are composed of cells, biomolecules as well as the extracellular matrix [1]. The extracellular matrix (ECM) is key in many developmental stages from embryogenesis to adult development, and tissue repair as well as the maintenance of tissue and organ homeostasis [1,2]. Once synthesized in the cytoplasm, ECM components are secreted into the extracellular space where they are then modified further into final molecules [1,2]. The main recognized function of the ECM is the provision of physical support for cells within tissues and organs as well as availing the transportation of biomolecules such as growth factors and cytokines to cells. Recent reports indicate that the ECM is involved in the activation of several mechanosensitive signaling cascades and therefore impacts several cellular processes [3,4,5,6,7]. The two forms of the ECM are the interstitial ECM and the basement membrane. This review mainly focuses on the interstitial matrix. 

**Figure 1:**
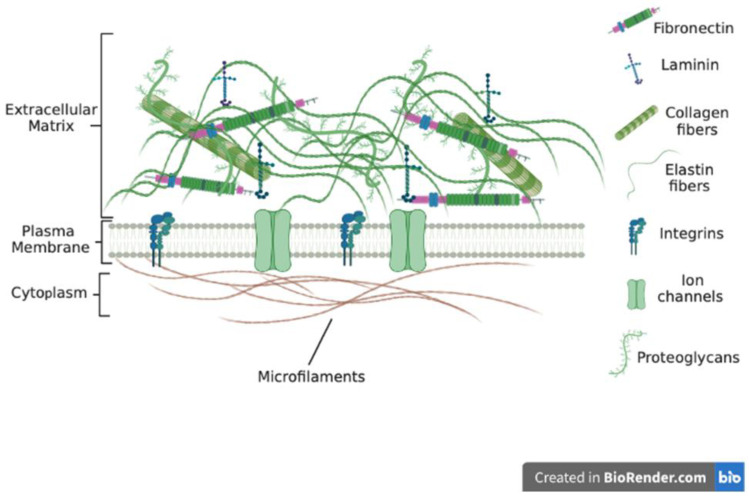
ECM molecules include collagens, laminins and fibronectin and proteoglycans. ECM is interconnected through various ECM molecules such as nidogen and perlecan. Proteoglycans bring collagen fibrils together to form large fibers.

The ECM is made up of several components that bond to form a complex network of different-sized molecules in a 3D unit (Figure 1). These ECM molecules are of different sizes, shapes, and spatial organization. Most tissues and organs have a specific type of ECM produced because of the differential expression of ECM genes as well as post-transcriptional splicing and post-translational modifications [8,9,10,11]. The ECM is in most cases in a state of flux, changing over time because of tissue development and disease [12,13,14]. Recent reports indicate that the ECM plays major role in disease progression and the development of chemoresistance [15,16,17,18]. Whilst cells synthesize the ECM, the ECM has been referred to as the ‘theatre’ within which cells interact with each other and biomolecules to effectively determine how cells behave [17,19]. Thus, cellular functions and phenotypes rely not just on gene expression but also on cues from the ECM. 

ECM remodeling under normal physiological conditions is a tightly controlled complex process, with many proteins playing different roles to maintain homeostasis. The ECM also undergoes remodeling during tumorigenesis, with several reports indicating that it can promote tumorigenesis as well as be antitumorigenic [20,21,22]. In the early stages of tumor formation, stromal cells synthesize large amounts of ECM proteins in a bid to protect normal tissue from tumor cells [17,23,24]. This results in the stiffening of the tissue around the newly formed tumor. The stiffening of the ECM is due to enhanced collagen as well as hyaluronic acid deposition [25,26,27]. Chronic insult to a tissue results in the enhanced synthesis of ECM proteins, leading to a ‘fibrotic’ condition. Reports indicate that enhanced ECM deposition is positively correlated with tumor initiation and growth [28,29,30]. Circulating tumor cells have been shown to hone and colonize tissues and organs displaying increased ECM synthesis [31,32,33]. Both ECM proteins and the biomolecules found within the ECM have been identified as valuable markers for the diagnostic analysis of tumors [34,35]. This review discusses ECM composition, function, and remodeling processes and presents evidence of several ECM components suggested as novel therapeutic targets and currently being investigated or undergoing validation [36,37,38,39]. 

## 2. The Extracellular Matrix Macromolecules

The macromolecules found within tissues as well as organs that surround cells and provide tensile strength and other cues are what is termed the extracellular matrix. Various ‘omics’ studies have comprehensively identified ECM components, referred to as the ‘matrisome’, and more than 200 genes have been assigned in humans [40,41]. The macromolecules form a fibrillar network that interacts with cells and biomolecules to influence cell behavior in tissues and organs. The exact number of extracellular matrix macromolecules in the human body is unknown. Two major classes of the ECMs are known: the tissue-specific ECM and interstitial ECM. The type and composition of the ECM vary depending on several factors including the tissue and organ of the body. There are several classes of ECM macromolecules including fibrillar collagens, filament-forming collagens and glycoproteins. Other classes include elastic proteins as well as proteoglycans. Important classes include the collagens that constitute the connective tissue. Under normal physiological conditions, the ECM is highly organized into sheets that confer tensile strength to tissues and organs. However, ECM composition may differ under conditions such as stress and diseases. The ECM also provides cues to cells via tethered biomolecules and ligands to effectively influence cell behavior [42,43]. 

## 3. Collagens

**Figure 2:**
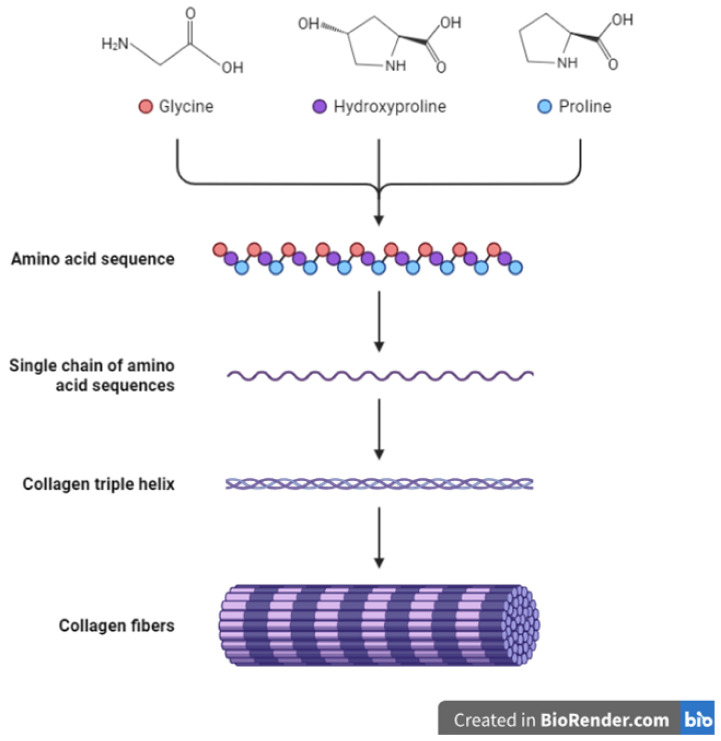
Schematic representation of collagen synthesis and structure.

The collagen family of proteins is the major component of the ECM and provides both mechanical strength and cues to cells and tissues. Reports indicate that collagens constitute around 90% of the ECM in humans [34,41]. Thus, collagens influence many cellular processes in the body including proliferation, migration, and adhesion [44]. Currently, about 28 proteins have been identified to belong to the collagen family [45]. Being the major proteins in the ECM, collagens undergo multiple changes and remodeling throughout an animal’s growth and development and in pathological conditions such as wound healing and cancers [46,47,48]. In addition, the synthesis of collagens requires modifications through the addition of disulfide bonds and other post-translational changes (Figure 2) [49,50]. Other ECM molecules also play a role in collagen synthesis and deposition. For example, the glycoprotein fibronectin is known to play a part in and influence the deposition and attachment of collagens in the extracellular space [51,52]. The overall structure and organization of the ECM are therefore results of the interaction between its constituents including collagens, glycoproteins, and other molecules [17,18,51,53,54]. Seven collagens have been grouped in the fibrillar class with type I collagen (or collagen type I) being a major component of this class. The other members include type II, type III, type V, type XI, type XXIV and type XXVII collagens [10,19,44,45]. Most collagens that form part of the basement membrane are grouped in the network-forming collagen class and these include type IV, type VIII, type X, type XV and type XVIII collagens [45]. Type VI and type XXVI collagens form the filament-forming class. The triple helical structure of some fibril-linked collagens can be interrupted and these include type IX, type XII, type XIV, type XVI, type XIX, and type XXII collagens [45]. Other collagens family members are found within or bound to membranes and these include type XIII, type XVII, type XXII, type XXIII and type XXV collagens [45]. 

Many studies have shown a link between changes in deposition and the amounts of collagens including the link between type I collagen and impaired development and the development of cancers [28,55]. Collagens found within the ECM in normal tissues can be highly uniform in orientation whilst in pathological conditions the orientation is varied [56,57]. Overall, the amounts of the different collagens in the ECM influence its properties from elasticity to availability of biomolecules such as growth factors and chemokines [58,59]. Collagens within the ECM also play other important roles within the body. For example, collagens are important within basement membranes where they contribute towards the separation of different layers of tissues. Increased collagen deposition within basement membranes can lead to membrane hardening disrupting the normal exchange of biomolecules and movement of cells [60,61]. In many pathological conditions such as cancer, basement membranes are thinner compared to normal tissues. This has been attributed to the reduced deposition of collagens including type IV, type XV, and type XIX collagens [62,63]. Indeed, several in vitro studies have also shown that collagen knockdown can enhance the migration of cancer cells [17,64,65]. 

## 4. Other Extracellular Matrix Macromolecules

A combination of proteins and carbohydrates make up glycoproteins and proteoglycans, with about 30 genes encoding these ECM components. The carbohydrates form repeating chains that are connected to a core made up of proteins. Proteoglycans are part of the glycoprotein family but are different from other glycoproteins in terms of their synthesis and structure. This ultimately influences their function in the body. Whilst glycoproteins have short and branched carbohydrate chains covalently linked to a protein core, the carbohydrate chains in proteoglycans are long and unbranched glycosaminoglycan chains also attached to a protein core [66,67]. Glycoproteins’ side chains create enough of a buffer to allow the ECM to resist stress and forces applied to the ECM [68,69]. In addition, glycoproteins are actively involved in regulating processes including proliferation and adhesion [69,70]. The glycosaminoglycan chains of proteoglycans are also negatively charged, allowing proteoglycans to impact the organization of other ECM constituents [70,71]. The negative charge of proteoglycans also allows the ECM as a whole to sequester growth factors and other biomolecules [72,73]. Due to their size and structure, proteoglycans can also participate in the binding of ligands to receptors, allowing cells to respond to various changes in extracellular cues. Several signaling pathways including the AKT-MEK and PI3-Akt cascades are activated through the participation of proteoglycans in bonding to various receptors [74,75]. The most well-known glycoproteins include fibronectin, fibrinogen, vitronectin, laminin, thrombospondins, periostin, and osteopontin. Among the well-known proteoglycans are decorin, aggrecan, and perlecan. 

### 4.1. Laminin 

A glycoprotein consisting of α, β, and γ chains that come together to form trimeric proteins, laminin or laminins is/are found within the basal lamina and contribute towards cell-specific functions including differentiation, adhesion, and migration [76,77]. Laminins as ECM glycoproteins play major roles in creating a link between the ECM and cells via binding to cellular receptors such as integrins. Thus, laminins are key to cellular migration and cancer cell invasive behavior. Currently, twelve mammalian chains (α, β and γ) have been identified, and these can combine in different amounts to form about sixty known laminins [78,79]. The α chains (200 to 400 kDa) are bigger than the β and γ chains (120 to 200 kDa) with the trimer being formed ranging from 400 to 800 kDa in size. Referred to as the ‘god molecule’ in some reports, trimeric laminin has a ‘cross’ shape formed as a result of its α-helical coiled-coil structure [80,81]. Laminins also bind to other ECM components including collagen type IV. In this case, laminins act as an intermediary or ‘glue’ between various ECM molecules within the basement membrane. Laminin polymerization is thought to be the main initiator of basement membrane assembly, placing laminin polymerization at the ‘center’ of cell function and tissue structure. 

A well-known laminin molecule is laminin-332 (LN-332), which is formed by β3, α3, and γ2 chains, plays key roles in cellular migration and adhesion and contributes to tumor cell metastasis [76,77,82]. Laminin molecules are also implicated in maintaining stem cell self-renewal capabilities. For example, laminin-332 maintains CSCs’ self-renewal abilities and contributes to drug resistance [83]. Several reports show that the presence of laminins is closely linked to significantly lower patient survival in cancers such as colorectal and pancreatic cancer [82,84]. Laminins bind to other ECM proteins, and this promotes cell migration and adhesion as well as enhancing drug resistance [85,86]. For example, the binding of laminin-332 to the integrin α3β1 receptor increases resistance to gefitinib in hepatocellular carcinoma [87]. Various signaling cascades are also known to be activated through laminin–integrin interactions. For example, laminins’ interactions with integrins the cause activation of the mTOR cell survival signaling pathway [83,88]. 

### 4.2. Fibronectin 

Structurally, fibronectin (FN) has several domains and is involved in the interactions between the ECM and cells. Fibronectin forms a fibrillar network and is key to cell differentiation, adhesion, and migration [89]. Fibronectin exists as a dimer of two molecules joined together via cysteine disulfide bonds. The assembly of fibronectin in the ECM occurs when it binds to α5β1 integrins via the RGD motif. Furthermore, the binding of fibronectin to integrins causes the clustering of integrin molecules, leading to increased levels of fibronectin molecules on the cell surface. Fibronectin-focal adhesion interactions alter the conformation of fibronectin, resulting in binding sites for other ECM molecules to be revealed. Fibronectin is therefore able to bind to collagens, laminins, and other proteins, allowing cells to adhere to the ECM and migrate [89]. Whilst it is a single gene-encoded protein, it has several isoforms resulting in proteins that form ECM fibrillar structures. Fibronectin binds to cell surface receptors and other ECM proteins such as collagens causing alterations to cells’ actin filaments, and this allows cells to migrate. Various reports show that fibronectin is key in cellular processes such as wound healing as well as in tumor growth [90]. Importantly, the adhesion of tumor cells to ECM proteins including fibronectin enhances the tumorigenic capacity of cancer cells as well as drug resistance [91,92]. Various studies have also associated increased fibronectin expression with tumor progression in various cancers [93,94,95]. Furthermore, clinical data associated enhanced fibronectin expression in tumors versus normal tissues with lower patient survival [90,96,97]. FN-induced migration was shown to be mediated via αvβ6 and α9β1 integrins in various cancers [90,98]. The binding of cancer cells to ECM proteins including fibronectin can protect cells from drug-induced apoptosis compared to cells attached to plastic [91]. Fibronectin-mediated reduction in apoptosis occurs via the inducement of cyclooxygenase-2 (COX-2) as well as the activation of integrin α5β1 [99,100]. In addition, various signaling cascades are activated when fibronectin binds to other ECM proteins [101]. The binding of cells to fibronectin also protects cells against many drug-induced states [83,102].

### 4.3. Periostin

Periostin is an adhesion-linked protein expressed as an ECM protein and produced within the periosteum as well as the periodontal ligaments [103,104]. It is a cell adhesion and nonstructural protein that functions to maintain tissue homeostasis, especially that of the tooth and bone tissues. It is mostly involved in many processes during development including cardiac development and healing but is expressed in low amounts in adult tissues [76,103]. Periostin mediates most of its effects via interacting with surface receptors such as integrins. The enhanced expression of periostin is associated with various pathological conditions including inflammation and disease, and many types of cancer including colon, lung, and breast cancer, and head and neck carcinomas [103,104]. Periostin is involved in regulating ECM–cell interactions via attachment to other ECM molecules including collagens, tenascin C, and fibronectin [104]. Periostin can bind to various integrins such as αvβ3, αvβ5, and α6β4, and thus influence the activation of many signaling cascades [105]. Some of the signaling cascades are Notch 1 and B-catenin signaling, which are important in cell differentiation and tissue specification. Various reports show that periostin is aberrantly expressed in pathological conditions such as arthritis, cancers, and fibrosis [104,105]. In various cancers, periostin has been shown to induce signaling cascades including PI3K-Akt through attaching to αvβ3 and αvβ5 integrins [106]. The presence of periostin enhances cancer cell proliferation and the process of EMT cancers such as gastric cancer [106,107]. Cancer cells showing resistance to various drugs including cisplatin and 5-fluorouracil (5-FU) also show increased periostin expression [104]. Thus, this suggests that increased periostin levels are correlated with drug resistance, tumor relapse, and tumor angiogenesis [108]. Periostin activates Akt phosphorylation in cancers including epithelial and ovarian cancer and carcinoma and this results in resistance, especially to paclitaxel [109]. Recent data suggest that periostin can be used as a prognostic marker in various cancers including pancreatic, ovarian, and esophageal cancers [110,111].

### 4.4. Hyaluronic Acid

Discovered almost a century ago by Karl Meyer and John Palmer whilst working on vitreous bovine eyes, hyaluronic acid is a glycosaminoglycan made up of *N*-acetylglucosamine and glucuronic acid repeats and is a common component of the ECM [112,113]. Hyaluronic acid is a long high-molecular-weight polymer with many hydroxyl moieties, allowing it to mix well in water [114,115]. Indeed, one of the main functions of hyaluronic acid is to retain water in various tissues [116,117]. Due to its size and the ability to form coils in water, hyaluronic acid can control the movement of biomolecules and ions within the ECM, allowing small molecules to pass whilst blocking the free movement or transport of larger biomolecules and substances [118]. Hyaluronic acid displays various unique properties such as biodegradability and great viscoelasticity, and has been utilized in various applications such as hydrogel formation and drug delivery systems [119,120]. Various studies have shown that hyaluronic acid plays crucial roles in cell migration and invasion through its interaction with receptors including CD44 and hyaluronan binding protein 4 [121,122]. 

## 5. Extracellular Matrix Function

The most important function of the ECM is providing an anchorage stage to cells as this is key to the maintenance of cell division and polarity. No longer is the ECM seen as only a scaffold necessary for cell structure, but also as a structure that provides both biophysical and biochemical cues to cells. In addition, the ECM can regulate cellular attachment and migration [123]. Several pieces of evidence also show that the ECM can sequester growth factors and other biomolecules, and these are released at specific stages of development and disease progression to influence cell behavior and phenotype [18,124]. During development, sequestered factors can cause gradients in biomolecule concentrations, and this is important during changes in tissue form and structure [124,125]. Furthermore, secreted factors are involved in the activation of various signaling cascades and influence focal adhesion formations [126,127]. 

The development of an organism from an embryo to an adult involves a lot of ECM changes, both in terms of quantity and type [128,129]. These changes must be controlled tightly at each stage of development to avoid overregulation and downregulation which can have deleterious effects. ECM physical properties including topography, elasticity, and rigidity influence cell proliferation and differentiation and ultimately influence tissue structure and integrity [7,16,53]. 

An important function of the ECM is that of aiding cell migration. For cells to migrate, their binding to the ECM via integrins and cadherins must occur first. Integrins allow cells to attach to various ECM molecules including collagens, fibronectin, and laminins. Integrins can then influence the intracellular actin cytoskeleton via focal adhesion proteins including talins and vinculins. ECM alignment and topography have been shown to influence both the speed of cell migration and the direction of migration [130,131]. To influence cell migration in a specific direction, scientists have utilized specific ECM molecules as well as ECM gradients [129,132]. Studies have shown that cells tend to migrate from low ECM concentration areas to areas of high ECM concentration, but this is not always the case [133,134]. The migration of cells is characterized by repeated adhesion to the ECM as well as deadhesion from the ECM [135]. Therefore, the rate of cell migration is dependent on various ECM properties including composition, alignment, and elasticity [136,137]. Our earlier publication demonstrated that cells migrate slower in ECMs lacking collagens compared to in collagen-containing ECMs [17]. ECM stiffness for example has been shown to influence cellular migration [138,139]. Investigations are under way to identify specific ECM components required for cell differentiation and migration during development as the ECM is constantly being remodeled. Importantly, for cells to migrate and invade surrounding tissues, the action of matrix metalloproteases and other proteases that can degrade the ECM is necessary [140,141]. 

The development of organs and tissues in the body is dependent on the presence of ECM proteins. For example, for the process of branching to occur, ECM proteins such as collagens and laminins are required to provide the anchor on which the formation of tubes can take place whilst ECM molecules such as hyaluronic acid allow epithelial cells to continue to migrate at the end bud [142,143]. The ECM’s alignment and architecture help in controlling tissue formation and branching patterns [142]. When a bud continues to grow, the ECM at the end is degraded, allowing various biomolecules including growth factors, cytokines, and chemokines to be released [140,144]. The released factors in turn influence the rate of branching and the direction of the bud [140,145]. The presence of the ECM at the end bud also means that various growth factors and signaling molecules can be sequestered and released at specific times when needed [69,146]. Whilst ECM proteins are important during budding, specific ECM proteins are required to stop the growth of the bud. For example, the deposition of type I collagen leads to the termination of the growth of the bud [142]. In summary, the ECM plays major roles during tissue and organ formation as well as in the formation of tubular structures. Importantly, in addition to the provision of a scaffolding on which tissues and organs can form, the ECM acts as a reservoir of various biomolecules needed by cells at specific times for differentiation and proliferation. 

Various studies have shown that the ECM induce cellular differentiation through the release of tethered factors as well as the physical structure and composition of the ECM [16,147,148]. To study the effect of ECM composition on cell fate, we cultured fibroblasts on a fibroblast-derived ECM (fd-ECM) and showed that these fibroblasts downregulated type I collagen synthesis compared to controls [16]. Via the use of function-blocking antibodies, our study demonstrated that the blockage of type I collagen gene expression in the presence of the ECM is mediated via integrins including α2β1 [16]. In addition, the same study revealed that ECM-mediated reduction in collagen was activated through the Ras-MEK/ERK signaling pathway [16]. Importantly, through a deletion analysis of the COL1A2 promoter, our study showed the presence of an ECM-responsive element within the -375 and -107 regions [16]. This study and other published reports demonstrate that ECM composition plays pivotal roles in determining cellular gene expression and function [149,150]. Using a different approach, we also investigated the fate of adipose-derived MSCs (ad-MSCs) when cultured on a cell-derived ECM (cd-ECM). The reason for use of the cd-ECM in this case was to model the in vivo physiological microenvironment. Our data showed that ad-MSCs cultured in the ECM lost their multipotency and differentiated into the chondrogenic cellular lineage compared to controls [7]. Elaborate studies including loss of function studies showed that cells can sense the mechanical environment and activate the Notch1 and β-catenin signaling cascades needed for ECM-mediated ad-MSCs chondrogenesis [7]. Thus, ad-MSCs must sense the mechanical environment of the ECM to undergo chondrogenesis, proving that the ECM can induce the differentiation of cells. 

The process of maintaining tissue homeostasis requires the ECM to be constantly altered as cells undergo proliferation and differentiation [151,152]. Once differentiated, cells must maintain their phenotypes whilst changes to tissues occur including the formation of new blood vessels [153,154]. A bidirectional interaction exists between cells and the ECM. In a classic study of the interaction between cells and the ECM, we demonstrated that cells can ‘feel’ the presence of ECM components and other ECM properties through integrins including α2β1 and adjust the gene expression of ECM proteins based on cues from the ECM [16,53]. Thus, a feedback process allows communication between the ECM and cells and is important to maintaining tissue homeostasis. Once this feedback mechanism is altered and unable to keep ECM degradation and deposition in check, conditions such as cancer can develop. Other ECM properties including elasticity also influence cell behavior. For example, Zhang and colleagues showed that ECM elasticity impacts then osteocyte gap junction elongation and also demonstrated the involvement of paxillin in signal transduction [155]. 

Various techniques have been utilized to study the role of the ECM during development and in tumorigenesis. The most common technique involves ECM gene knockout, downregulation, and upregulation, leading to the alteration of ECM composition [156,157]. The addition of enzymes that can degrade specific ECM proteins can also be used to alter ECM composition and reveal the role of specific proteins in various cellular processes [158,159,160]. Antibodies against ECM proteins and their respective receptors including integrins can be used to investigate the role of ECM proteins in the development and maintenance of homeostasis. In our earlier publication, we showed that function-blocking antibodies used to downregulate collagen gene expression revealed that type I collagen interacts with α2β1 integrin [16,48]. Other techniques that can be used to study ECM proteins’ role in development and tumorigenesis include the use of a three-dimensional culture, atomic force microscopy, and ECM protein crosslinking [7,161,162]. 

Studying the composition of a tissue and organ ECM is challenging due to its complexity. ECM molecules are large and undergo further modifications, such as hydroxylation, glycation, and glycosylation [163]. To profile ECM composition, various methods, and assays have been developed, including SDS-PAGE, to separate ECM proteins, followed by HPLC and mass spectrometry [164,165]. Choosing the ideal sample preparation and identification steps are crucial for successful ECM analysis [166]. Tailored experimental and analytical approaches are needed to decipher the complexity of the ECM with ECM molecules displaying different solubilities and post-translational modifications [167]. Various methods have been used to solubilize the ECM, ranging from the use of 5 M Guanidine-HCL to 8M urea and 100mM DTT [164,168]. The challenge lies in solubilizing ECM proteins without degrading them, and different methods can be used, ranging from the use of harsh solutions to the use of gentle solubilization.

To obtain more ECM proteins, various methods can be used for sample preparation, such as the use of different-strength detergents, enzymatic digestion, or a photocleavable detergent called azo which can be used in the fractionation approach to characterize the ECM composition of tumors [168,169]. There is no best method for ECM sample preparation, and scientists often use less harsh solutions to identify many ECM proteins. ECM proteomics has adopted methods used for global proteomics, such as TMT and iTRAQ for the accurate determination of protein abundance, and peptide fractionation using high-pH reversed-phase LC [170,171]. Methods such as data-dependent acquisition are the major methods used for ECM profiling, and recent studies have also employed data-independent acquisition [172,173]. To accurately identify and estimate protein abundance, it is important to maximize peptide-to-spectrum matching as well as the use of standards [174,175,176]. Most importantly, various resources are available for scientists in ECM research. These include various databases and websites such as the MatrisomeDB (https://matrisomedb.org/ (accessed on 23 October 2022)), TopFIND (https://topfind.clip.msl.ubc.ca/ (accessed on 12 June 2022)), MatriNet (https://www.matrinet.org/ (accessed on 20 August 2022)) and The Manchester Peptide Location Fingerprinting website (https://www.manchesterproteome.manchester.ac.uk/#/MPLF (accessed on 8 May 2022)).

## 6. Extracellular Matrix Modifications

The composition of the ECM in a specific location or tissue is influenced by several parameters such as the synthesis and degradation of its components. In addition, the biomolecules found within the ECM are location- or tissue-specific and are influenced to a large extent by resident stromal cells such as fibroblasts and immune cells. Further changes in ECM composition and the biomolecules found within it are brought about by the action of enzymes including matrix metalloproteases and hydroxylases [177,178,179]. Studies in various cancers including breast and bladder cancers have shown that the post-translational hydroxylation of collagens leads to increased crosslinking and is linked to low patient survival [180]. The crosslinking of ECM components, sulfation as well as glycosylation are some of the further changes that occur to ECM components after synthesis [177,178,179]. These post-translational changes impact ECM components’ interactions with other members of the ECM as well as with receptors on cell surfaces [7,16,53]. Whilst changes in ECM composition and sequestered biomolecules are necessary for tissue homeostasis, the state of lax is also necessary for development and growth. Altered synthesis and accumulation of any one component of the ECM can alter the existing delicate process of homeostasis and lead to conditions including fibrosis and the promotion of cancer growth [181,182,183,184]. The contribution of stromal cells and immune cells has been recognized as key to the maintenance of homeostasis and the development of several pathological conditions [17,18,185,186,187]. 

Reports indicate that when there is enhanced crosslinking of the ECM, a dense meshwork of ECM components is formed, leading to fibrosis and other pathological conditions [29,153,188,189]. Importantly, the accumulation of ECM proteins leads to stiffening, which influences ECM-receptor interactions and cellular signaling [29,190,191]. Excessively crosslinked ECM proteins also lead to reduced ECM turnover, allowing some ECM proteins to prolong their presence within certain tissues. For example, ECM proteins known to promote wound healing via their participation in certain stages of the process may prolong their presence around the wound, leading to an aberrant process [192,193,194]. Various enzymes are known to take part in ECM crosslinking, and these include lysyl oxidases, and transglutaminases [195]. The lysyl oxidase family of enzymes allows the deposition and accumulation of collagens and elastins in the ECM, and this has significant implications for cell morphology and movement [196,197]. By influencing the deposition of collagens in the ECM, lysyl oxidases also affect cellular signaling and the response to therapy [198]. Transglutaminases are enzymes involved in glutamine deamination during ECM protein and glycoprotein synthesis [199,200]. Transglutaminases are also involved in transamidating glutamine residues during ECM synthesis and the proper alignment of fibers during ECM synthesis. The alignment of fibers by transglutaminases leads to ECM stiffening and a reduction in proteolytic degradation. A stiff ECM influences ECM-receptor interactions including integrin-mediated signaling [201]. Another form of spontaneous ECM crosslinking is glycation, a process including Amadori rearrangement and Schiff base adduct formation. This process does not involve enzymes. 

The glycosylation of ECM molecules has been linked to various processes of tumorigenesis. For example, the enhanced glycosylation of fibronectin leads to increased EMT and high levels of invasive cell behavior in prostate cancer cells and carcinomas, respectively [202,203,204]. The inhibition of integrin glycosylation including that of αvβ6 integrin leads to the enhanced invasive behavior of cells involved in metastasis [205,206]. Demonstrating the importance of fibronectin as a component of the ECM, the phosphorylation of fibronectin leads to increased mechanical forces being needed for cell adhesion in various cancers [207,208,209]. Glycosaminoglycans can undergo sulphation in various cancers, and this impacts cell–matrix signaling [210,211,212]. 

## 7. Proteolytic Degradation of the Extracellular Matrix 

Homeostasis involves the constant synthesis and degradation of ECM components over time [153,213]. Enhanced or reduced synthesis and degradation beyond what is normal can lead to several pathological states including fibrosis. Many enzymes are involved in both the synthesis and degradation of the ECM. Importantly, ECM degradation is controlled by zinc-containing endopeptidases including matrix metalloproteinases and a disintegrin and metalloproteinase proteins with thrombospondin motifs (ADAMTSs) [214,215]. Excessive ECM degradation can lead to an abnormal ECM characterized by unbalanced ECM components or ECM components that are not crosslinked properly [216,217]. The occurrence of more ECM degradation than synthesis can result in the removal of whole tissue components such as that of basement membranes and the vasculature, allowing cells to migrate in an uncontrolled manner [218,219,220]. The levels of growth factors and cytokines previously bound to ECM proteins can increase locally due to excessive ECM degradation, leading to the unregulated activation of signaling cascades [17,43,221]. For example, TGF-b and VEGF have been shown to be released from degraded ECM proteins through the action of MMPs, leading to the activation of various signaling cascades and angiogenesis [18,222,223,224]. Inorganic ions including calcium ions can also be released from the degraded ECM, leading to the activation of calcium-dependent MMPs [225]. 

One major class of enzymes involved in ECM degradation is that of matrix metalloproteinases (MMPs). As reviewed by Kesenbrock and colleagues, human MMPs are made up of a total of 23 enzymes with a Zn-containing domain as well as four hemopexin-like domains [226]. Four MMP members, namely MMP14, MMP15, MMP16 and MMP24, contain both a transmembrane and cytoplasmic domain. Through degrading the ECM, MMPs impact cellular processes via the release of sequestered growth factors and cytokines [227]. Whilst most MMPs have a specific substrate leading to their grouping as collagenases and gelatinases for example, many other MMPs cannot be grouped this way [186]. Due to their actions in the body, MMPs are highly regulated to ensure the maintenance of homeostasis as well as to allow growth and development. The unregulated expression and the eventual action of MMPs have been associated with many pathological conditions [228,229,230]. For example, MMP2 and MMP11 are associated with poor survival in ovarian cancer [231]. However, some MMPs including MMP8 have been associated with increased survival in oral squamous cell carcinoma patients [232]. The varied and sometimes opposing actions of MMPs in the body require derailed efforts to develop inhibitors for these enzymes. 

There are 21 disintegrin and metalloproteinase proteins (ADAMs) and about 19 ADAMs with thrombospondin motifs (ADAMTSs) in humans that play a role in degrading the basement membrane of vascular system vessels [233,234]. Many ECM proteins and proteoglycans including collagens, fibronectin and vitronectin are degraded by ADAMs [234]. ADAMTSs are secreted enzymes and involved in degrading proteoglycans and collagens [140]. Several signaling cascades have been shown to be influenced by Adamalysins through the removal of ligands from the cell surface [235,236,237]. 

Cathepsins are a family of ECM-degrading enzymes made up of 11 serine, cysteine, and aspartic peptidases [238,239,240]. Cathepsins are mostly found in lysosomes and involved in the degradation of protein precursors such as procollagens within the cell [187,241,242]. Cathepsins have been implicated in altered homeostasis and many pathological conditions including scar formation and cancer development and metastasis [243,244,245,246]. On the contrary, cathepsin B is important during tissue regeneration in wounded human epidermal keratinocytes [247]. 

Originally referred to as procollagen C-proteinases, bone morphogenetic protein I and tolloid-like proteinases are enzymes that play key roles in the maturation of procollagen molecules and have no known role in ECM degradation [248]. These enzymes can cleave the carboxy terminus of procollagens, leading to the maturation of the procollagen molecules into collagens [248,249]. These proteinases work in cahoots with growth factors in promoting ECM deposition during growth and development [250,251]. Bone morphogenetic protein I and tolloid-like proteinases are essential in skin wound healing but their upregulation is linked to corneal scarring [252,253]. 

Hyaluronidases are involved in the degradation of hyaluronan in the body to maintain tissue homeostasis. Hyaluronan is rapidly degraded after synthesis compared to other ECM components, and this is essential for tissue homeostasis [254,255]. The accumulation of hyaluronan as well as its increased degradation are often associated with several pathological conditions including cancers [255]. Additional enzymes, CEMIP and transmembrane protein 2, are also referred to as hyaluronidases, and the increased activity of hyaluronidases can lead to the formation of hyaluronan fragments that have been linked with the increased formation of blood vessels [256,257]. Hyaluronan fragments, resulting from enhanced hyaluronan degradation, have also been linked to the increased synthesis and release of chemokines and cytokines, leading to the activation of various signaling cascades [258,259,260]. Reports indicate that hyaluronan fragments accumulate during injury and lead to increased inflammatory factors being synthesized by immune cells around a wound [261,262,263]. The persistent inflammation occurring as a result of the lack of removal of hyaluronan and its fragments can lead to the promotion of tumorigenesis [264]. Dysregulated hyaluronan production disrupts the normal ECM structure as well as the formation of blood vessels [265]. The main hyaluronan receptor is CD44 and it is involved in the removal of hyaluronan and its fragments in case of injury. 

Heparin sulfate glycosaminoglycans (HSGs) are cleaved by heparinase from proteoglycan core proteins and eventually degraded into oligosaccharides [266]. HSGs are involved in ECM organization and the activation of cell signaling via their binding to other ECM components as well as receptors. Heparanase has been associated with enhanced wound healing and angiogenesis in various animal models [267,268]. However, its overexpression has been linked to other pathological conditions including cancers [269,270]. The disruption of normal heparinase expression causes ECM–heparan sulfate interactions to be altered, leading to weak ECM structures. Cell movement can be increased under these conditions as ‘pores’ are present in the ECM, and these allow cells to migrate easily. 

To maintain a normal ECM structure and the number of components, ECM-degrading enzymes are tightly regulated by the action of their respective inhibitors. Inhibitors of ECM degrading enzymes are secreted by various cells, and they act in both autocrine and paracrine modes. Some of the well-known inhibitors of ECM-degrading enzymes are the tissue inhibitors of metalloproteinases and cystanins. One of the well-known endogenous inhibitors of MMPs is the tissue inhibitors of the metalloproteinase (TIMP) family. In mammals, this family has four members, namely TIMP1, TIMP2, TIMP3, and TIMP4 [271]. TIMPs have recently been shown to inhibit the Adamalysin family of ECM proteinases, expanding their role in maintaining ECM homeostasis [272,273]. Disruption in TIMP synthesis and secretion has been noted in many pathological conditions including cancers and aberrant wound healing [274,275]. 

The action of cathepsins is reversibly inhibited by cystatins. Cystatins have both intracellular and extracellular activities and therefore influence both ECM synthesis and the remodeling of mature ECMs. Cystatins are also known to show inhibitory activities against papain and legumains [276]. Serine and cysteine proteases involved in the degradation of various ECM proteins and proteoglycans can be irreversibly inhibited by serpins [277]. Serpins play a significant role in maintaining tissue and vascular homeostasis as well as in fibrinolysis [278]. Serpins have also been shown to play key roles in thrombosis [279]. Serpins are part of a large family and therefore have many contrasting roles in mammals. Several ECM molecules contain structures called cryptic domains that release fragments called ‘matricryptins’ when the molecules are cleaved [280,281]. These ‘matricryptins’ are important for cell adhesion and differentiation [140]. Due to their many functions, some ‘matricryptins’ may serve as enzyme inhibitors and abrogate proteolytic activities, thus influencing ECM synthesis and degradation [281]. One ‘matricryptin’ derived from the collagen XVIII molecule is Endostatin which has been shown to cripple the function of androgen receptor [282]. Included in the matricryptins derived from collagen are arresten, tumstatin and canstatin, and these have been associated with many pathological conditions [283,284,285]. Several matricryptins have been shown to have antigrowth properties and can cause senescence in cells, but evidence also points to the fact that a ‘matricryptins’ fragment from ECM molecules such as laminin 111 can promote cellular growth [286]. 

## 8. Fibroblasts and Extracellular Matrix Remodeling

**Figure 3:**
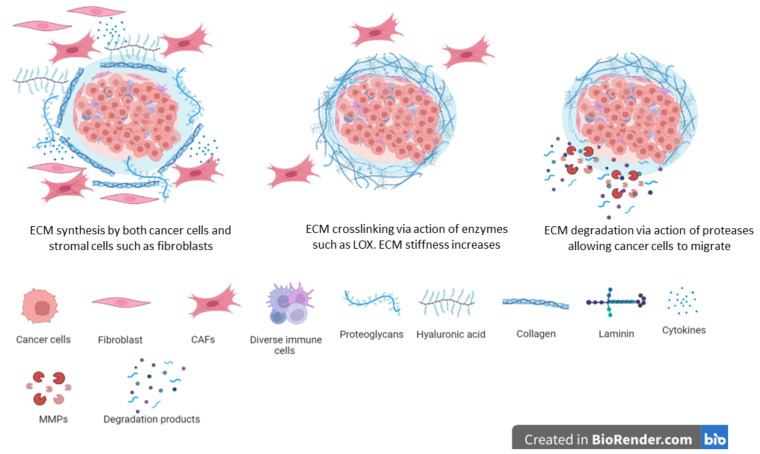
Extracellular matrix remodeling in tumors. Normal resident fibroblasts within tissues are activated and transform into cancer-associated fibroblasts via the release of factors by tumor cells. CAFs synthesize large amounts of ECM around the tumor. ECM-modifying enzymes including MMPs cause release of matrikines that are pro-tumorigenic.

The unregulated synthesis and deposition of the extracellular matrix which is associated with many cancers results in fibrosis or the so-called ‘hardening’ associated with mostly advanced tumors (Figure 3) [29,287]. Whilst all stromal cells contribute towards the synthesis and deposition of the ECM, cancer-associated fibroblasts are the main cells carrying out this job. In addition, recent reports indicate that cancer cells also synthesize and deposit ECM proteins and proteoglycans [17,18,288]. Similarities have been noted between the cancer-associated fibroblasts and fibroblasts found during wound healing [289,290]. Cancer-associated fibroblasts display great heterogeneity and different cells have been suggested as sources of CAFs [291,292]. Both local and recruited cells are potential sources of CAFs. For example, local fibroblasts can easily be recruited to growing tumors where they undergo the activation of CAFs. Mesenchymal stem cells from the bone marrow as well as adipose tissue are transformed into CAFs [18,293]. The activation of local fibroblasts and transformation of other cells into CAFs are driven by growth factors and chemokines released by both cancer cells and stromal cells [18]. Such growth factors and chemokines can be released within the vicinity of the cells or can be transported via exosomes from distant environments [294,295]. 

Studies show that the presence of CAF presence and the increased synthesis and deposition of ECM are linked to the recurrence of disease and decreased patient survival [296,297,298,299]. Research into which subset of CAFs drive tumorigenesis and the recurrence of disease is needed as great heterogeneity is displayed by CAFs, with some subsets of CAFs being known to be involved in inflammation whilst others being known to be myofibroblast-like, for example [300,301]. Attempts have been made to characterize and classify CAFs, but the jury is out on the utility of such endeavors. In addition to the phenotypical differences displayed by CAFs, their precise location in the body and tumors also determine the role they play in various processes. Thus, CAF subsets release different growth factors and chemokines depending on their spatial location. Data are lacking on the changeability of the different CAF subsets and the contribution of the different subsets to tumorigenesis and disease outcomes. Since CAFs, other stromal cells and cancer cells contribute to the ECM and biomolecules found within tumors, it is currently difficult to assign a specific role to CAF-derived, stromal cell- and cancer-derived ECM molecules in disease progression and outcomes. Cancer cells within tumors for example have also been shown to synthesize unusual ECM proteins and proteoglycans that may play a role in fibrosis and disease progression [302,303,304]. 

Hyperactivation of the sympathetic nervous system due to cellular stress is associated with the remodeling of the stroma [305,306,307]. Furthermore, cellular stress has been shown to promote the synthesis and release of ECM molecules such as collagens [308]. Increased ECM synthesis is linked to the development of chemoresistance in various cancers [17,309,310]. For tumor cells to migrate and metastasize, there is need for space and the secretion of factors involved in invasion. ECM deposition is downregulated by tumor cells via the release of colony-stimulating factor 1 [311,312,313]. Reduced ECM synthesis leads to enhanced tumor cell invasion and migration. The specific contribution of ECM molecules, from either tumor cells or stromal cells to the progression of tumors is still a subject of intense research. In addition, the specific point when ECM molecules change from antitumorigenic to pro-tumorigenic ones and vice versa is not yet known. Another approach is to ‘normalize’ stromal cells involved in ECM synthesis, so that there is a normal production of ECM components [17,18]. 

## 9. Extracellular Matrix Signaling 

Tumorigenesis leads to alterations of the ECM in terms of structure and composition, with such alterations being pro-tumorigenic [314,315,316]. Furthermore, such ECM alterations can also result in the development of chemoresistance [317,318]. Cues from the ECM can be physical as well as sequestered biomolecules and these cause immediate as well as long term changes in gene expression [319,320]. The turnover period of most ECM proteins and glycoproteins can be hours, days and weeks long and thus their continued presence provides a continuous stimulus to cells, leading to the activation of cellular cascades over a long time. 

Whilst stromal cells and tumor cells can both release growth factors and chemokines and thus influence signaling, the ECM can enhance or decrease the resulting signaling via the release of sequestered growth factors and chemokines and by sequestering synthesized factors, respectively [315,316,317,318,319]. In addition, the stiffness of the ECM can influence integrin-based signaling during normal development and in diseases [321,322]. Reports indicate that signaling cascades including the MEK-ERK and the JNK signaling cascades can be activated by ECM stiffening in various conditions [323,324,325]. Other signaling cascades also respond to ECM composition and stiffness, thus targeting these signaling cascades together with targeting ECM composition and stiffness are plausible strategies to control tumorigenesis and metastasis. 

One of the major receptors of ECM-cell interactions is integrins. Integrins are heterodimers involved in transmitting extracellular cues into cellular signaling [16,326]. During development and in some pathological conditions, specific integrins are expressed and these influence specific cellular activities such as migration, proliferation and adhesion [327,328,329]. Importantly, the binding of various integrins including αvβ1, αvβ3 and α4β1 to ECM molecules has been linked with tumor cell invasion during tumorigenesis [330]. The precise expression of certain integrins may be linked to the promotion of tumorigenesis, drug resistance and metastasis [331]. The enhanced expression of α5β1 and its binding to the ECM molecule fibronectin has been linked to reduced drug efficacy in models of cancers [332,333]. Overall, the involvement of integrins in development and pathological conditions such as cancers depends on the type of integrin, ECM molecules and cell type [334,335,336]. The interconversion of integrins has also been associated with ‘cadherin switching’ during the epithelial to mesenchymal transition [337,338]. The switching or conversion of integrins from one heterodimer to another is thought to be linked to their binding to different ECM ligands as well as to different cells [339]. 

Several nonintegrin receptors are known to bind to ECM components and these include heparan sulfate proteoglycans bound to the surface of cells, the discoidin domain containing receptor 1 as well as leukocyte-associated immunoglobin-like receptor 1 [340,341]. Together with integrins, these receptors relay extracellular cues and signals to activate various signaling cascades. The activation of these nonintegrin ECM receptors in stromal and cancer cells can enhance ECM synthesis via a feedback loop, thereby decreasing the access of drugs to cancer cells [342,343,344]. For example, syndecan 4 is highly expressed in various cancers and is known to activate signaling cascades associated with cancer cell survival [345,346]. A major receptor for collagen and hyaluronic acid is CD44. Structurally, CD44 traverses the cell membrane and has both an extracellular domain and a cytoplasmic component. The interaction of CD44 and hyaluronic acid leads to the activation of various other receptors including EGFR and c-MET [347,348]. Through the activation of signaling cascades, hyaluronic acid-CD44 plays a key role in tumorigenesis [349,350]. The infiltration of lymphocytes into tumors is partly mediated by the interaction of CD44 with fibronectin, which allows lymphocytes to bind to fibronectin and other ECM components [351]. CD44 has been reported to play key roles in tumorigenesis and its overexpression is linked to poor patient survival and drug resistance [352,353]. CD44 is one of the important cancer stem cell markers in many cancers including colon cancer, breast cancer, prostate cancer, and lung cancer [354,355,356,357]. 

External cues are sensed by receptors on the cell membrane and converted into cellular signaling to cause specific cellular behavior. This process is known as mechanotransduction and is important in various cellular processes, from those of normal development to those in diseases. Mechanotransduction influences all cells within tumors, ultimately determining the progression of tumor development [358,359]. Whilst cells influence the deposition and accumulation of components of the extracellular matrix, extracellular matrix properties such as those of a mechanical nature and elasticity modulate cell behavior [360,361,362]. Various signaling cascades are known to be activated by extracellular matrix-derived cues and these include MEK-ERK, PI3K-Akt and YAP-TAZ signaling [363,364]. 

Cellular behavior is modulated not just by the amount of ECM components, but also by the biochemical properties of the ECM such as tensile strength, mechanoresistance, and elasticity. These biomechanical properties affect cellular processes such as cellular migration and metabolism [365,366]. The adhesion of cells to the ECM of different elasticity shows that ECM elasticity influence gene expression and integrin levels on cell surfaces [367]. In addition, cues and signaling molecules released during the adhesion of cells to the ECM influence the organization of the cytoskeleton and therefore affect cellular migration and invasive behavior [368,369]. Various signaling cascades including FAK, and PI3K-Akt have been shown to modulate ECM–cell interactions, influencing normal cell growth and movement [370,371,372]. An increase in ECM proteins including collagens has been associated with the migration of cells and the development of chemoresistance [373,374]. 

## 10. Extracellular Matrix and Cell Invasion and Metastasis

**Figure 4:**
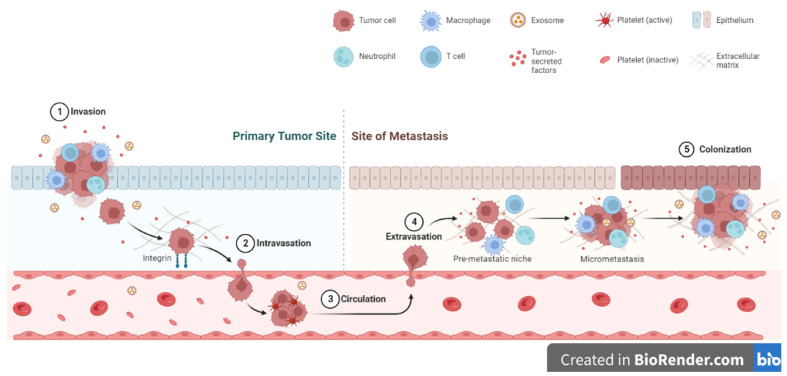
The role of the ECM in tumor metastasis. Increased MMP activity around and within the primary tumor leads to tumor cells intravasating into blood vessels. Tumor cells can be protected by ECM during circulation. ECM molecules including fibronectin aid circulating tumor cells to attach to endothelial walls leading to extravasation of tumor cells into distant sites already ‘prepared’ for the arrival of tumor cells. These sites are called ‘premetastatic sites’. Resident cells within premetastatic sites including fibroblasts synthesize large quantities of ECM molecules and release various growth factors that support metastasis formation.

Four major features of metastasis are the migration of cancer cells from their origin, their honing to new sites and the regulation of secondary sites in preparation for tumor growth, the heterogeneity of cancer cells and lastly the colonization of the new sites and growth of secondary tumors (Figure 4) [19,375,376]. At each stage of the metastatic process, the ECM plays a central role and its remodeling influences the progression of the process. The alignment of ECM proteins including collagens and fibronectin has been shown to influence tumor cell metastasis in various cancers [377,378,379,380,381,382]. Linear collagen molecules appear to promote tumor cell migration as the spaces between fibers allow cancer cells to move in a certain direction [377,378,379,380]. Furthermore, the action of both tumor- and stromal cell-derived MMPs in the degradation of ECM molecules allows spaces to be created for tumor cell migration and invasion into surrounding areas [383,384,385]. ECM molecule post-translational modification processes including hydroxylation also promote metastasis by promoting the enhanced synthesis of ECM molecules [179,386,387]. Due to their size and physical structures, ECM molecules can shield invading and migrating tumor cells from the effects of shear stress during the tortuous journey to secondary sites [386,387,388]. One major feature of successful metastasis is the preparation of ‘new sites’ for tumor cells to colonize and grow. Various theories have been given to explain this process. For example, it is thought that tumor-derived MMPs degrade and remodel the existing ECM of the ‘new sites’ before tumor cells colonize these sites [389,390]. Following the ‘seed and soil’ hypothesis, this remodeling of the existing ECM in new sites is important to create the right pro-tumorigenic environments for colonization by metastatic tumor cells [391,392]. Overall, it is accepted that the ECM plays an important role in allowing metastatic tumor cells to colonize new sites and be able to grow. In other instances, tumor cells do not grow into secondary tumors but remain quiescent for some time. These tumor cells can survive for a long time, reawaken, and grow into tumors after a long time [393,394,395,396,397]. For example, the upregulation of the ECM molecule periostin has been shown to promote the reawakening of breast cancer cells from dormancy [398,399]. 

## 11. Extracellular Matrix in Drug Resistance: The Extracellular Matrix Shields Tumor Cells from Anti-Cancer Drugs

**Figure 5:**
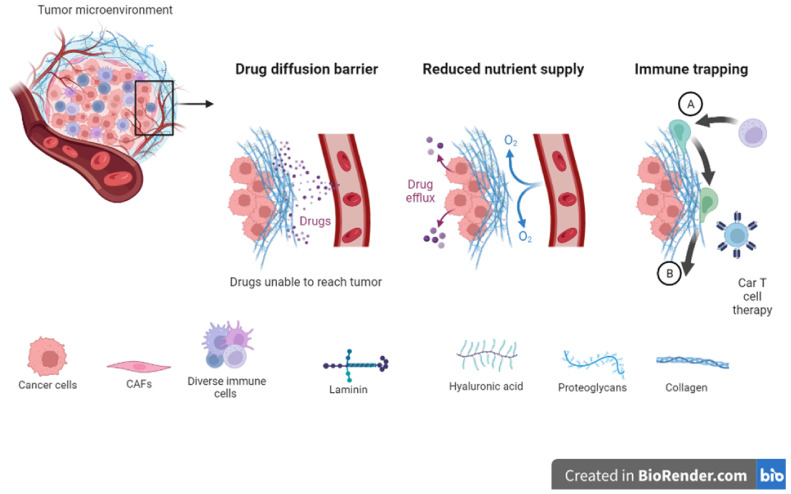
Tumor ECM reduces therapeutic efficiency in solid tumors. ECM components interfere with drugs reaching tumor cells and thus prevent therapeutic effects. Large quantities of ECM around tumors lead to less effective concentration of drugs reaching tumor cells. Reduced oxygen leads to hypoxic conditions within tumor microenvironment and this causes tumor cells to activate drug release mechanisms and drug resistance pathways. The physical barrier formed by ECM also means that immune cells cannot access tumor cells.

Studies reveal that the ECM is a major player in tumorigenesis and treatment outcomes [400]. Furthermore, reports indicate that therapy itself can induce ECM remodeling and can result in molecule deposition within tumors. TGF-B levels have been associated with increased ECM remodeling induced by drugs [401,402]. Increased levels of endogenous tissue inhibitors of metalloproteinases (TIMPs) are linked to positive clinical outcomes in many cancers [403,404]. On the other hand, increased levels of receptors including integrins are linked to poor outcomes and disease recurrence [330,405,406]. ECM stiffness impacts the adhesion of cells, movement of cells, and response to therapy [407,408,409,410,411]. Increased matrix stiffness within tumors is linked to less responsive tumors and drug resistance (Figure 5) [388,412,413]. Generally, stiffer ECMs are found surrounding tumors compared to ECMs in normal tissues [410,414,415,416,417,418,419,420]. ECM stiffness is linked to fibrosis in many cancers including breast cancer where it is observed that many signaling cascades are also activated [316,323,421,422,423,424]. Furthermore, various reports indicate that a stiff TME promotes tumor progression via the activation of integrin signaling [425,426,427,428]. Tumor metastasis is promoted by ECM stiffening via the action of lysyl oxidase and deposition of collagens [429,430,431]. ECM stiffening can also induce microRNAs involved in the downregulation of the tumor suppressor protein PTEN [432,433]. Drug delivery relies on diffusion and pressure within the interstitial spaces [434]. The remodeling of the ECM can create a barrier to drug diffusion and either impede drug movement altogether or limit its movement and therefore reduce its effectiveness [435,436,437]. 

The question on every scientist’s mind is whether or not the ECM is of relevance to disease initiation and progression beyond what is already known. An analysis of tumor biopsy samples has so far revealed normal and disease-specific ECM signatures in various cancers [438,439]. Low levels of specific ECM molecules including decorin have been associated with poor patient’ survival in various cancers [303,440,441,442]. The understanding of ECM composition and the amounts of components in it at different stages of tumorigenesis is valuable in disease targeting. Drugs can be designed to target or have great adherence to specific ECM molecules to deliver the drugs to specific tumor sites [443]. Studies in various cancers have shown that specific cancers have a specific ECM signature that is predictive of patient survival [303,439,444], and the question is whether or not specific ECM proteins are pro-tumorigenic or antitumorigenic. Various in vitro studies have shown that the knockdown or removal of certain ECM proteins can sensitize cancer cells to drugs [17,445,446]. Currently, the use of cancer-specific ECM signatures in treatment strategies is limited and requires a further detailed analysis of the ECM proteins at specific stages of tumorigenesis. Data from Senthebane and colleagues suggest that targeting fibrillar collagen and fibronectin in tumors may allow drugs to access tumor cells and therefore improve therapeutic efficiency [17]. 

Many approaches have been utilized to block the pro-tumorigenic properties of the ECM. For example, stopping the expression of collagens and fibronectin leads to reduced drug resistance of cancer cells [447]. Several ECM members are known to accumulate in various cancers, and their degradation in combination with chemotherapy can result in better outcomes for patients [448,449]. Furthermore, since most ECM proteins require post-translational modifications for stability and to have the right conformation, the disruption of modifications can also result in unstable ECM proteins. This has a two-fold effect: a decrease in ECM components leads to less fibrosis and can lead to increased drug efficacy and reduced ECM components lead to fewer MMPs being required and present within the TME. The presence of fewer MMPs can reduce the aggressiveness of many cancers [450,451,452]. It is important to note that inhibitors of MMP activities have given very disappointing results in many clinical trials [453,454,455]. Part of the reason why MMP inhibitors did not succeed as expected is the presence of many members of the MMP family and their overlapping activities [456,457]. 

In addition to targeting ECM components, many signaling cascades that are activated or downregulated by ECM proteins can be regulated. Various survival pathways are activated in various cancers [458,459,460]. Many developed small-molecule inhibitors can abrogate cancer cell signaling, leading to induced drug sensitivity in cancer cells [461,462]. Whilst studies using inhibitors of ECM synthesis and signaling give promising data, it is important to note that ECM components can have both pro-tumorigenic and antitumorigenic behavior. This complex behavior requires deep analysis, and many hurdles are still to be overcome. 

## 12. Therapeutic Strategies Targeting the Extracellular Matrix 

Considerations of ECM composition as well as of genetic mutations have allowed the administration of drugs that target specific ECM molecules and specific organs, leading to improved patient outcomes. Important in this regard are new drugs that target specific ECM components, either to upregulate or downregulate their expression, as these can be used in combination with existing drugs. The total removal of specific ECM components may not be the best way forward as this can promote tumor progression and impact normal tissue function. In tumors, collagen levels can be regulated at different stages of their synthesis and degradation. Collagen levels can be controlled by targeting various signaling cascades involved in their synthesis such as TGF-B signaling. Antibodies including fresolimumab are currently under clinical trials in cancers where they are used to reduce collagen levels [463]. The inhibition of TGF-B signaling through the use of halofuginone is effective at reducing collagen levels in various cancers [464,465]. Other drugs used to target the TGF-B cascade include pirfenidone, metformin, tranilast and Ki26894 [466,467]. Caution is needed when inhibiting collagen levels via blocking the TGF-B signaling cascade as TGF-B is involved in other body processes such as inflammation [468,469]. Another way to reduce collagen levels is to use collagenases. In normal tissues, collagenases can easily be made available to degrade collagen [470]. In solid tumors that are compact, collagenases cannot be transported easily due to their large size [471]. A major issue regarding degrading collagen within solid tumors is the potential release of sequestered growth factors, resulting in unintended effects [472,473]. In addition, the degradation of any ECM component may create ‘highways’ for cancer cell migration and metastasis [44,474]. MMPs can also be used to degrade ECM components such as collagen. The effectiveness of MMP use and of the use of their inhibitors in cancers has been disappointing with many clinical trials yielding no good results [475]. 

Various cancer therapies have been used to target fibronectin and these include its potential as a drug delivery molecule. Reports indicate that fibronectin or its isoforms are upregulated in many cancer tissues as well as in normal tissues [476,477,478]. Antibodies against fibronectin domains including L19 have been used to inhibit cancer cell growth [479,480]. In addition, peptides that bind to the fibronectin domain EDB (extra domain B) can be used to deliver drugs and drug-containing exosomes to tumors resulting in the better shrinkage of tumors than can be achieved with just drugs alone [481]. Another ECM protein that has been targeted during cancer therapy is hyaluronic acid. Hyaluronic acid synthesis can be inhibited by 4-methylumbelliferone. The inhibition of hyaluronic acid synthesis leads to the loss of tissue integrity, thus causing tumors to be leaky with no proper structure. Hyaluronic acid synthesis inhibition thus leads to more drugs reaching tumor cells compared to tumors with normal hyaluronic acid levels [482,483]. Hyaluronic acid can also be degraded via the use of hyaluronidase. Various reports and clinical trials are underway to evaluate the usefulness of hyaluronidase in combination with drugs [448,484]. Various integrins, expressed by cancer cells, can also be targeted in various cancers. The use of antibodies against integrins has shown great results in cancers including breast and colon cancers [485,486]. Such anti-integrin antibodies include volociximab and vitaxin. Small-molecule integrin antagonists can target specific integrin–ECM interactions that can block integrin-mediated cancer cell migration and therefore prevent tumor cell invasion and metastasis. Such integrin antagonists include cilengitide [487,488]. Antibodies against CD44 including bivatuzumab and RO5429083 have shown antitumor activity in patients with advanced cancers [489,490]. 

ECM-targeted therapy involving biomimicry is a type of treatment that aims to mimic the natural structure and function of the ECM. Biomolecules and biomaterials are designed to replicate properties of the ECM such as its composition, mechanical properties and signaling cues. A common approach to ECM biomimicry involves using natural ECM components such as collagen, fibronectin, and laminins as building blocks for synthetic materials. A combination of these ECM components can create materials mimicking various natural ECM structures and functions. ECM biomimetics for cancer therapy has been used in the development of ECM-based scaffolds for drug delivery [491]. The scaffolds are designed to mimic the structure and composition of a natural ECM and can therefore deliver drugs directly to tumor cells [492]. The ECM-based scaffolds provide a physical barrier that protects drugs from possible degradation and clearance during circulation and thus allows drugs to reach tumor cells at the right concentration [493,494]. This increases drug efficacy as well as reducing systemic toxicity. Knowledge of the ECM surrounding tumor cells can lead to tumor-specific drug uptake and enhances drug retention at the tumor site for more effective cancer treatment [495,496]. 

ECM-based scaffolds can be used to support the growth and differentiation of stem cells which have been shown to differentiate into cell types including those that can destroy tumor cells [497,498]. Stem cells can also be used to deliver anticancer drugs directly to tumor cells as well as generate new tissues to replace tissues damaged due to cancer or therapy [499,500,501]. ECM mimicking scaffolds can also induce brown adipogenesis in mesenchymal stem cells, which have been shown to have potential antitumor properties [502,503,504,505]. A bioinspired nanofiber scaffold mimicking the ECM structure of the spinal cord can lead to the regeneration of an injured spinal cord, a common side effect of cancer treatments [506,507]. A promising area of ECM biomimetics for cancer therapy is the development of ECM-mimicking nanoparticles for targeted drug delivery [508]. Nanoparticles can be designed to mimic the structure and function of the ECM, enhancing their ability to selectively target cancer cells expressing certain receptors or proteins [509,510]. The nanoparticles can be loaded with therapeutic agents including small interfering RNAs (siRNAs) that selectively knockdown or knockout genes involved in tumor growth and metastasis [511,512]. By targeting cancer cells only, ECM-mimicking nanoparticles can improve drug efficacy and reduce systemic toxicity [513]. 

Furthermore, researchers are exploring the use of ECM-mimicking hydrogels for the delivery of immunotherapeutic agents [514]. Immunotherapy has emerged as a promising approach in cancer treatment, stimulating the patient’s own immune system to recognize and attack cancer cells [515]. ECM-mimicking hydrogels can be used to deliver immunotherapeutic agents, such as checkpoint inhibitors, directly to the tumor microenvironment, enhancing its ability to stimulate an immune response against cancer cells [516,517]. Finally, ECM biomimetics is being studied for the development of new imaging techniques for cancer diagnosis and treatment monitoring [518]. By mimicking the structure and composition of the ECM, these imaging techniques can provide high-resolution images of the TME, allowing clinicians to monitor both tumor growth and the response to therapy [518,519]. This information can guide treatment decisions and optimize cancer treatment outcomes.

## 13. Conclusions

The ECM exists in normal and tumor tissues. During development, the ECM performs functions including providing structural support for cells and directing cell differentiation. In tumors, the ECM is needed for giving continual support to the growing tumor mass as well as to promote tumor cell migration and metastasis. Over the years, new technologies and bioinformatic softwares have been developed to delve deeper into the composition of both normal and tumor ECMs, revealing that the ECM can be used in directing cellular function and in a diagnostic and predictive manner, for example. A better understanding of the ECM has led to efforts being made to interfere with its synthesis and degradation to improve patient outcomes. In its simplistic nature, targeting individual ECM proteins inadvertently affects other physiological processes and must be investigated further. Disruptions to ECM synthesis and degradation will impact tissue homeostasis, a complex state maintained by many interlinked processes. Importantly, merely disrupting ECM synthesis and degradation is not going to stop pathological conditions such as cancer from developing but is required to be combined with therapeutic strategies such as chemotherapy, immunotherapy, and radiotherapy. ECM biomimetics has the potential to revolutionize cancer therapy, providing new strategies for drug delivery, stem cell-based therapies, targeted immunotherapy, and cancer imaging. This calls for further deeper investigations of how multiple antitumor strategies can be combined to have synergistic or additive effects. 

## Data Availability

Not applicable.
